# Gradual and selective trace-element enrichment in slab-released fluids at sub-arc depths

**DOI:** 10.1038/s41598-019-52755-9

**Published:** 2019-11-08

**Authors:** Simona Ferrando, Maurizio Petrelli, Maria Luce Frezzotti

**Affiliations:** 1 Department of Earth Sciences, Università di Torino, Via Valperga Caluso 35, 10125 Torino, Italy; 2 Department of Physics and Geology, Università di Perugia, Piazza Università 1, 06100 Perugia, Italy; 3 Department of Earth and Environmental Sciences, Università di Milano-Bicocca, Piazza della Scienza 4, 20126 Milano, Italy

**Keywords:** Geochemistry, Petrology

## Abstract

The geochemical signature of magmas generated at convergent margins greatly depends on the nature of fluids and melts released during subduction. While major- and trace-elements transport capacity of ultrahigh pressure (UH*P*) hydrous-silicate melts has been investigated, little is known about solute enrichment and fractionation in UH*P* (>3.5–4 GPa) solute-rich aqueous fluids released along colder geothermal gradients. Here, we performed *in situ* LA-ICP-MS trace-element analyses on selected UH*P* prograde-to-peak fluid inclusions trapped in a kyanite-bearing quartzite from Sulu (China). The alkali-aluminosilicate-rich aqueous fluid released from the meta-sediments by dehydration reactions is enriched in LILE, U, Th, Sr, and REE. Inclusions trapped at increasing temperature (and pressure) preserve a gradual and selective trace-element enrichment resulting from the progressive dissolution of phengite and carbonate and the partial dissolution of allanite/monazite. We show that, at the investigated *P-T* conditions, aqueous fluids generated by dissolution of volatile-bearing minerals fractionate trace-element distinctly from hydrous-silicate melts, regardless of the source lithology. The orogenic/post-orogenic magmas generated in a mantle enriched by metasomatic processes involving either solute-rich aqueous fluids or hydrous-silicate melts released by the slab at UH*P* conditions can preserve evidence of the nature of these agents.

## Introduction

Hydrous fluids and silicate melts-released by dehydration or melting of subducting slabs at sub-arc depths^1–7^-are able to fractionate trace elements and to generate the chemical signature of orogenic and post-orogenic magmas, i.e. high contents of both large-ion-lithophile elements (LILE; e.g., Rb, Cs, Ba) and light-rare-earth elements (LREE), and low contents of high-field-strength elements (HFSE)^8,9^. Trace-element systematics based on experiments and natural rocks indicate that hydrous-silicate (HS) melts generated by high-pressure (H*P)* and ultrahigh-pressure (UH*P)* dehydration melting along high-temperature gradients are very efficient metasomatic agents, enriched in LILE, LREE, Th, U^10–18^ by at least one order of magnitude with respect to aqueous fluids released by sub-solidus dehydration reactions along low-temperature gradients^10,19–25^. At intermediate geothermal gradients, aqueous fluids are released at *P* and *T* near or just above the second critical end-point of the H_2_O–pelite and H_2_O–granite systems (ca. 750 °C; 3.0–3.5 GPa)^26,27^. At these conditions, fluids have physicochemical properties intermediate between aqueous fluids and hydrous-silicate melts, and their solute load increases dramatically even for small temperature rise by progressive mineral dissolution^22,26^. The only, semi-quantitative trace-element data from a natural UH*P* aqueous fluid released during deep subduction (730 °C; 4.0–4.3 GPa) show strong enrichment in LILE, LREE, Pb, Sr, U, and Th^28^, i.e. enrichments similar to that observed in fluids produced by experiments at *P-T* conditions above the second critical end-point of H_2_O–silica(te) systems^11,14,29,30^. Whereas some authors^11,13^ suggest that there are no significant differences in trace-element content and fractionation between aqueous fluids (including supercritical fluids) and HS melts generated at similar temperature conditions, others^30^ argue that the UH*P* aqueous fluids have higher capacity than melts to mobilize LREE. The fluid evolution, as dissolution processes move forward, is also poorly constrained.

Here, we report the trace-element composition of a natural, UH*P* “alkali-aluminosilicate-rich aqueous fluid” (hereafter “aqueous fluid”) released from a meta-arenite subducted at *P* > 3.5 GPa along an intermediate *P-T* gradient. The composition was obtained by *in situ* Laser Ablation Inductively Coupled Plasma Mass Spectrometer (LA-ICP-MS) analyses on multiphase-solid inclusions trapped in kyanite during UH*P* prograde-to-peak evolution of a kyanite-bearing quartzite from Sulu (China).

This study allows characterizing, for the first time, the gradual and selective enrichment of trace elements in a natural aqueous fluid released by prograde-to-peak dissolution of phengite, carbonate and allanite/monazite from subducted UH*P* meta-sediments. Dissolution processes occurring in the investigated *P-T* range generate solute-rich aqueous fluids (probably above the second critical end-point of the H_2_O-pelite system^27^) with similar trace-element fractionations (e.g., Th/La, La/Ta), regardless of the involved lithology. These fractionation patterns are distinct from those in HS melts produced by partial-melting processes. Potassic-ultrapotassic magmatism can preserve the trace-element fractionations of the subduction-related metasomatic agent, with implications on the geodynamics of convergent margins.

### Multiphase-solid inclusions petrography and chemical composition

Studied fluid inclusions occur as multiphase-solid inclusions (MSI’s) in UH*P* porphyroblastic kyanite of a kyanite-quartzite from Hushan (Donghai area, southern Sulu terrane, China). The Qinling-Dabie-Sulu orogen formed by Triassic subduction and collision of Yangtze craton beneath Sino-Korean craton. In southern Sulu terrane, the UH*P* Unit consists of orthogneiss with minor paragneiss, eclogite, amphibolite, ultramafic rocks, quartzite, and marble^31^. Peak metamorphic conditions, dated at 235–225 Ma^32^, are primarily estimated at *T* = 730–890 °C and *P* = 3.5–4.5 GPa^31,33,34^, although lower *P-T* conditions are also suggested (Supplementary Information). Weakly-foliated kyanite-quartzites occur within gneiss and consists of pre-kinematic MSI-rich porphyroblastic kyanite, grown from phengite during UH*P* prograde-to-peak path, retrograde syn-kinematic inclusion-free kyanite, and OH-rich topaz, muscovite, paragonite, and pyrophyllite. Rutile, zircon, pyrite, barite, monazite, and apatite are accessory minerals. The sample for fluid inclusion study (RPC547) was selected among those used to constrain metamorphic and fluid evolution of kyanite quartzites^34,35^ (Supplementary Information).

**Figure 1:**
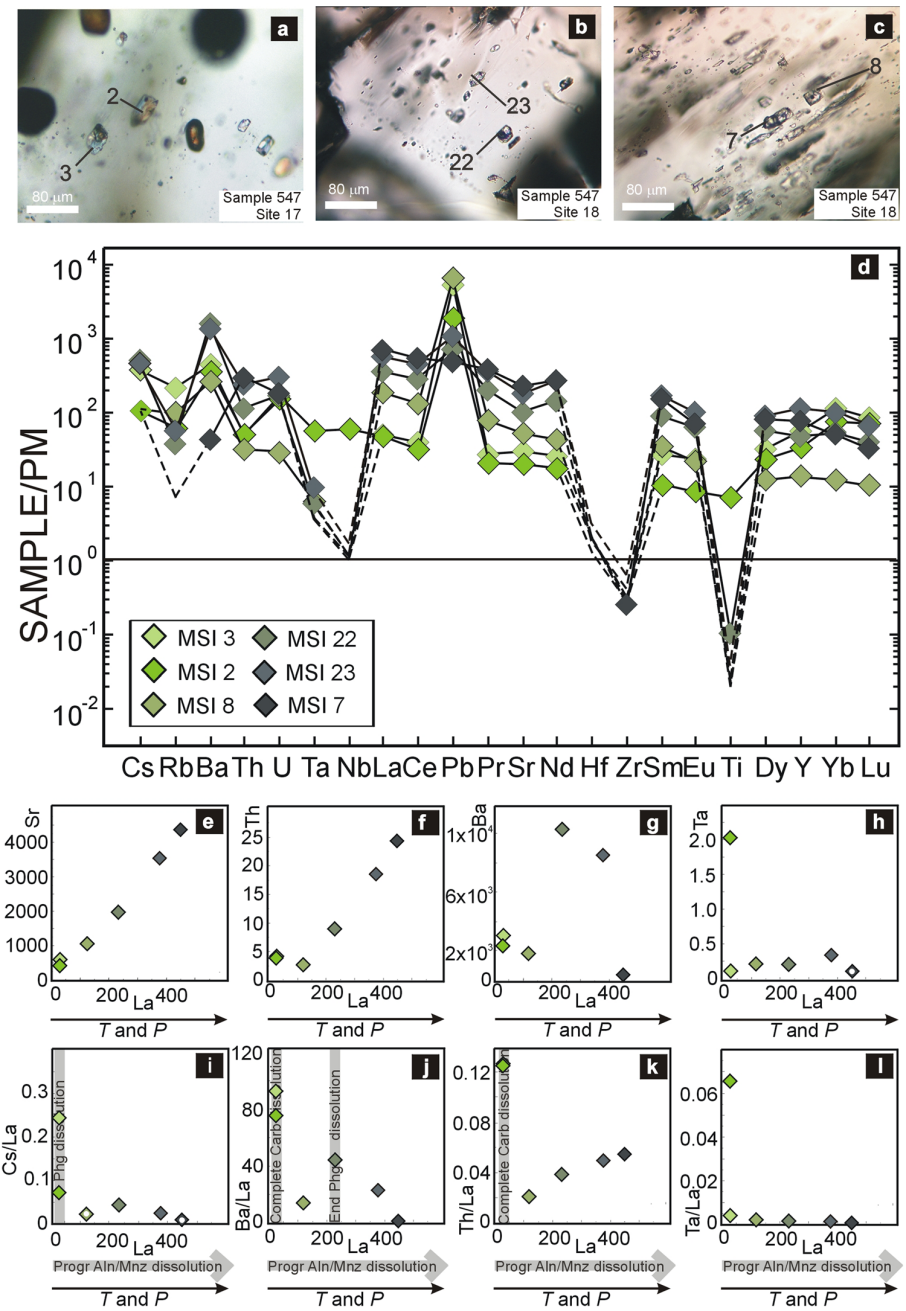
Analyzed MSI’s and their trace-element distribution and ratio. **(a**–**c)** photomicrographs of the analyzed MSI’s (plane-polarized light) with the related analysis number (see Supplementary Table 2). **(d)** Trace-element patterns are normalized to the primitive mantle^48^. Data below detection limits are plotted as detection limit values, without symbols and connected with dashed lines. **(e**–**h)** Trace-element concentrations (in ppm) with respect to La. Note the progressive increase in the La content from MSI’s from the prograde kyanite core (MSI2–3) to the peak kyanite rim (MSI7). **(i**–**l)** Diagrams showing the variation in trace-element ratios with respect to La (in ppm) in the fluid produced by progressive/complete dissolution of carbonate, phengite and allanite/monazite during UH*P* prograde-to-peak evolution. The white dot refers to ratios in which the element detection limit has been used as maxim element content.

Studied MSI’s are primary inclusions evenly distributed in UH*P* core and rim of porphyroblastic kyanite (Supplementary Information). Selected MSI’s have negative-crystal shapes and dimensions variable from 5 to 30 μm in length (Fig. 1a–c; Supplementary Fig. 3). They lack evidence for post-trapping modifications^26^ and contain muscovite, paragonite, K-Na-hydrous sulfate, anhydrite, carbonates, minor pyrite, barite, corundum, and water. Solid and liquid phases show relatively constant proportions and represent the daughter minerals precipitated from the trapped fluid and the residual liquid H_2_O, respectively^26^. More rarely, MSI’s may contain zircon and/or rutile of variable size that were incidentally-trapped during kyanite growth. The average major-element composition of MSI’s indicates that the aqueous fluid is alkali-aluminosilicate-rich, containing Al, Si, S, Ca, Fe, Mg, K, Na, and CO_2_ (Supplementary Table 1).

The trace-element composition budget of the aqueous fluid has been recalculated by mixed (MSI + host kyanite) data obtained by *in situ* LA-ICP-MS analyses (see Methods; Supplementary Table 2). The trace-element patterns (Fig. 1d) of analyzed MSI’s are relatively homogeneous and display significant enrichments in REE, LILE, U, Th, Sr, and Y with respect to the primitive mantle, with exceptions of Nb, Hf, Zr, and Ti. The local enrichment in Ta, Nb, and Ti measured in one MSI (MSI2) (Fig. 1a–d) is probably due to very-small rutile grain just below the MSI or incidentally trapped in it.

Fractionation patterns show systematic differences between MSI’s trapped in porphyroblastic kyanite core and those trapped in the rim. Whereas MSI’ in the inner core (MSI2, 3; Fig. 1a–d) have patterns with relatively lower enrichments in LREE, MREE, Th, Sr, and strong enrichments in LILE (Cs, Ba, Rb), Pb and U, the most external (kyanite rim) MSI7 (Fig. 1c,d) shows significantly higher enrichment in LREE, MREE, Th, Sr and lower enrichment in Pb, Rb, Ba. The MSI8, 22, and 23, located in the outer core (Fig. 1b,c), show intermediate patterns (Fig. 1d) for LREE, MREE, and Sr. The MSI8 has the highest enrichment in Pb and the lowest in U and Th, whereas MSI22 and 23 have the highest enrichment in Ba.

The concentrations of Sr, Th, Ba, and Ta relatively to La are reported in Fig. 1e–h. Lanthanum and other LREE contents (e.g., Supplementary Fig. 4a) are relatively low in MSI2 and 3, and steadily increase from MSI2 and 3 to MSI8, 22, 23, 7 (i.e., from the prograde core to the rim). A similar trend is also observed for Sr (Fig. 1e) and MREE (Supplementary Fig. 4b), whereas Th (Fig. 1f), Ba (Fig. 1g) and HREE (Supplementary Fig. 4c) show distinct behaviors. The thorium content steadily increases only in MSI8, 22, 23, and 7 (Fig. 1f), whereas Ba content considerably increases in MSI23 and 22 and sharply decreases in MSI7 (Fig. 1g). HREE are relatively high in MSI2 and 3, whereas MSI8 has a very little HREE content that gradually increases in MSI22 and 23, and it decreases in MSI7. Noteworthy, in the inner core of kyanite, MSI2 and 3 show lower LREE, Sr, and Th contents than MSI7 in the kyanite rim; in the outer core, MSI22 and 23 have the highest Ba contents. The Ta content, such as the other HFSE, is constant and always very low (Fig. 1h), except for MSI2 (see above).

### Trace-element release by progressive mineral dissolution in UH*P* aqueous fluids

Our data on MSI’s indicate that, at the investigated *P-T* conditions (ca. 3.5–4.0 GPa; 750–850 °C), slab dehydration reactions involving phengite in meta-sediments can release alkali-aluminosilicate-rich aqueous fluids from the slab. In agreement with previous studies^28,34,35^, UH*P* aqueous fluids could have been generated near the second critical end-point of the H_2_O-pelite system. Present data reveal that these fluids are extremely enriched in LILE, U, Th, Sr, and REE, but not in HFSE and that they progressively change the amount and fractionation of their trace-element budget during UH*P* prograde-to-peak *P-T* evolution.

According to experimental data^22,25^ and thermodynamic models^36^, if dehydration reactions occur near or just above the second critical end-point of the H_2_O-silica(te) system, even a small change in *T* ± *P* ± pH can produce relevant variations in the amount and kind of solutes because the rock-forming minerals steadily dissolve in it due to their increasing solubility in water. Thus, both the nature of rock-forming minerals and their degree of dissolution are responsible for the gradual variations in trace-element content and fractionation. Considering the very simple UH*P* mineral assemblage of the studied lithology, the HFSE negative anomalies measured in MSI’s (Fig. 1d–h) indicate that rutile and zircon do not contribute to the fluid trace-element budget. Conversely, the substantial increase of Ba, a compatible element in phengite, in MSI22 followed by its sharp decrease in MSI7 (Fig. 1g) suggests the complete exhaustion of phengite during UH*P* prograde metamorphism, in the absence of any other suitable residual host phases for Ba at the UH*P* metamorphic peak. The observed enrichment trends in REE, Th, and Sr (Fig. 1e,f; Supplementary Fig. 4) argue for the contribution of an accessory mineral containing these elements, undetected during optical observation. Suitable minerals are apatite, monazite or allanite, but not xenotime, being this last one uncommon in eclogitic rocks^37–41^. Apatite can be ruled out because it grows during retrograde evolution (Supplementary Fig. 2b). Aggregates of monazite locally occur in quartzites from Hushan (Supplementary Fig. 2f), but the lack of phosphates as daughter minerals in studied MSI’s seems to be in contrast with its contribution to the fluid chemical budget. Alternatively, a contribution could be provided by allanite, that usually occurs in many H*P*–UH*P* lithologies, in particular in meta-impure quartzites^37,40^ (Supplementary Fig. 5).

Selected trace-element ratios plotted relatively to La (Fig. 1i–l; Supplementary Fig. 6) allow better defining their release processes during the gradual dissolution of their host minerals. Phengite hosts Cs, Rb and Ba (Supplementary Fig. 5) and, at the beginning of its destabilization, Cs preferentially partition to the fluid whereas Ba remains in phengite until its complete dissolution; Rb has an intermediate behavior^15,28^. The high Rb/La ratio and the moderate Cs/La ratio recorded in MSI3, located in prograde kyanite core (Fig. 1i; Supplementary Fig. 6a), indicate that this early-formed inclusion trapped an aqueous fluid released during progressive phengite dissolution. Similarly, both the sharp decrease of the Ba/La ratio (Fig. 1j) in prograde MSI23, after a definite increase in MSI22, and the extremely low Ba/La ratio measured in MSI7, located at the peak kyanite rim, reveal that the peak fluid is released after complete phengite consumption. As an interesting consequence, the relatively high Ba contents in both MSI2 and 3 (Fig. 1g) could indicate dissolution of minimal volumes of another mineral that also released Sr, Th, and HREE (Fig. 1e,f; Supplementary Fig. 4c). Its complete dissolution during prograde conditions is testified in particular by the high Ba/La, Th/La, U/Th, Sr/La, U/La, ratios (Fig. 1j,k; Supplementary Fig. 6d–f). The most plausible candidate is a carbonate^42^, as also suggested by the presence of carbonates in MSI’s (Supplementary Fig. 3j,k). Carbonate could have partitioned Ba with phengite (Supplementary Fig. 6b) and Sr, Th, U, and REE with allanite/monazite (Supplementary Fig. 6c–f). Allanite and monazite host LREE that behave like major elements during mineral dissolution (Fig. 1d,e). A similar behavior is exhibited by Sr (Fig. 1d,e) probably due to the lack of other major Sr-bearing minerals, e.g. omphacite (Supplementary Fig. 5), in association with allanite/monazite in studied lithology. Moreover, allanite/monazite and carbonate dissolution paths mainly control the HREE and Y fluid budgets, because the studied quartzite does not contain garnet.

Thus, the progressive variations in trace-element ratios from analyzed prograde to peak MSI’s indicate the complete dissolution of carbonates and phengite in the presence of dissolving allanite/monazite. The lack of allanite/monazite in the rock matrix suggests that the process could have continued up to their complete dissolution.

### The metasomatic efficiency of UH*P* aqueous fluids

**Figure 2:**
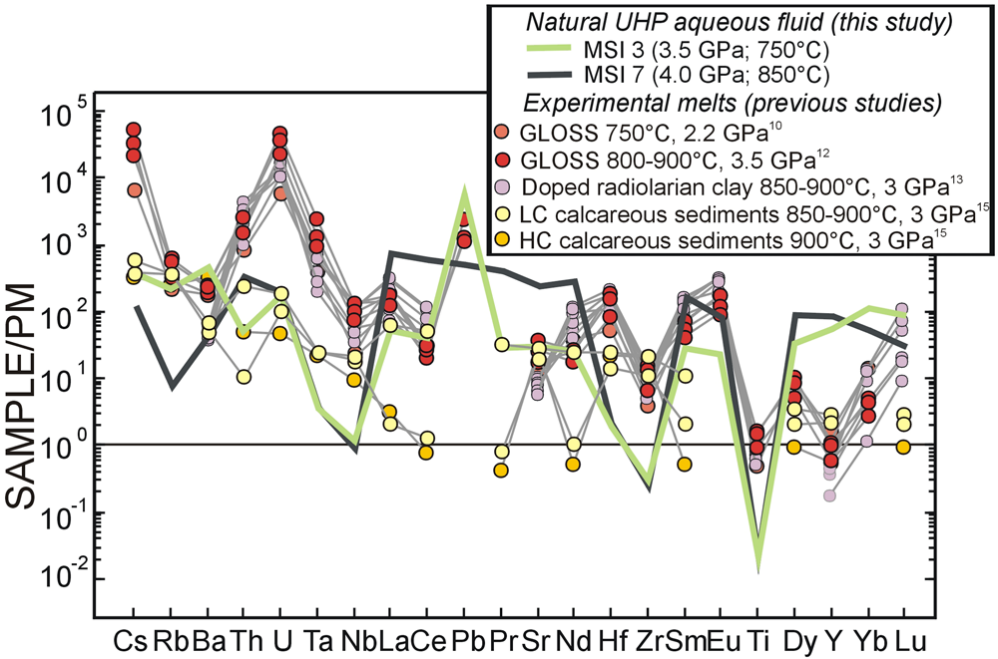
Comparison between trace-element patterns of the analyzed natural UH*P* aqueous fluid and the experimental hydrous-silicate melts released by sediments at high *P-T* conditions. Trace-element concentrations are normalized to the primitive mantle^48^. Data below detection limits are plotted as detection limit values. LC: low carbon; HC: high carbon.

The typical trace-element patterns of the analyzed UH*P* aqueous fluid (trapped at ca. 3.5–4.0 GPa and 750–850 °C) can be compared with those of HS melts obtained at similar *P-T* conditions from experiments^10,12,13,15^ (Fig. 2). Trace-element patterns of HS melts from artificial metapelite and radiolarian clay are very similar to each other and strongly enriched in Cs, Th, U, Ta relatively to the primitive mantle. These melts are also enriched in all the other incompatible elements, except for Ti and Y. These patterns diverge from those obtained from natural calcareous clay and are characterized by lower enrichments in Cs, Th, U, Ta, and REE. The trace-element fractionation pattern obtained at 900 °C from a low-carbon calcareous sediment is similar to those obtained in this study, in particular to the pattern of the prograde (3.5 GPa and 750 °C) fluid (MSI3).

**Figure 3:**
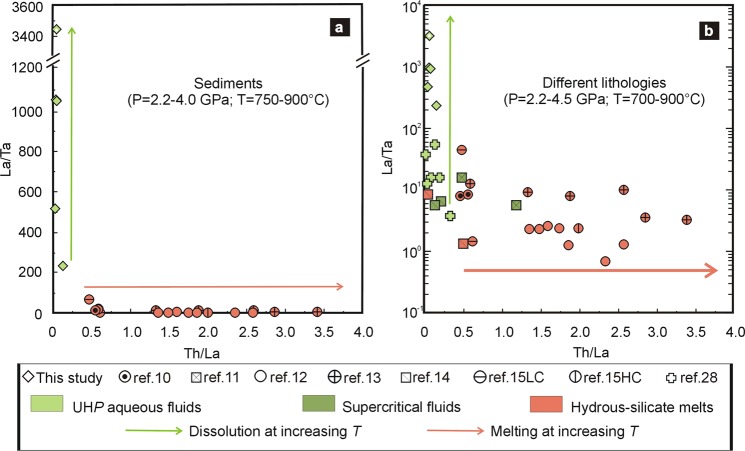
Diagnostic diagram to distinguish the nature of slab-derived fluids. **(a)** Th/La vs. La/Ta variations in the analyzed natural UH*P* aqueous fluid and in experimental hydrous-silicate melts released by sediments at high *P-T* conditions. **(b)** Th/La vs. La/Ta variations in natural UH*P* aqueous fluids and in experimental supercritical fluids and hydrous-silicate melts released by different lithologies at high *P-T* conditions. MSI2 was not plotted because its Ta content is disguised by the presence of a mineral not precipitated from the fluid (see text). LC and HC: low-, and high-carbon calcareous sediments.

Interestingly, the LREE enrichment of the peak (4.0 GPa and 850 °C) fluid (MSI7) is one order of magnitude higher than that in the experimental melts. Our work indicates that aqueous fluids released from meta-sediments along intermediate subduction gradients at pressures greater than 3.5 GPa can transport similar or even higher amounts of trace elements (in particular LREE) than HS melts generated from similar lithologies at UH*P*, in agreement with recent experiments on allanite-bearing eclogite^30^, but incorporates lower amounts of HFSE. As evident in Fig. 3a, studied UH*P* aqueous fluids are characterized by low Th/La and by a gradual increase in La/Ta with temperature. Conversely, experimental melts from meta-sediments are characterized by low La/Ta and a gradual increase in Th/La with temperature. These two distinct fractionation trends suggest a different behavior of the same minerals (i.e., rutile, zircon, allanite/monazite, and carbonate) during dissolution or partial-melting processes at the considered *P-T* conditions. For example, rutile and zircon do not dissolve in UH*P* aqueous fluids, whereas they participate to melting at increasing temperature (Supplementary Fig. 7).

Aqueous fluids released at pressure above 3.5 GPa have likely attained conditions near or just above the second critical end-point of the H_2_O-pelite system^27^. The possibility to distinguish between these “supercritical” aqueous fluids and hydrous-silicate melts by their trace-element fractionations is still debated^11,13,30^. In Fig. 3b, natural and experimental data on UH*P* “supercritical” fluids and HS melts released from different lithologies (e.g., metasediments, metabasics, Mg-metasomatic rocks) are plotted in a Th/La vs. Ta/La diagram. As evident, it seems to be a promising diagnostic diagram to identify the nature of a metasomatic agent released at the considered range of *P-T* conditions (700–900 °C and 2.2–4.5 GPa), regardless of the source lithology. At *P-T* conditions higher than those investigated, the complete incorporation of minerals in both aqueous fluids and HS melts makes these two metasomatic agents chemically indistinguishable from each other, including their trace-element content. It is noteworthy that this overlapping, observed in some experiments^11,13,14^, occurs at *P-T* conditions distinct from a lithology to another, since the P-T conditions of the second critical end-point depend on the considered chemical system.

Supercritical fluids are considered more mobile than HS melts because at *P-T* conditions near or just above the second critical end-point the structure of the solutes changes from (hydrated) ions to monomers and higher polymers^43^. In particular, their viscosity is lower than that of HS melts, enhancing element-transport capacity from the source (e.g., slab) into the surroundings (e.g., overlying mantle wedge)^25,44^. The presence of LREE and LILE-rich UH*P* veins containing quartz ± omphacite (or jadeite) ± kyanite ± allanite ± zoisite ± rutile ± garnet precipitated from supercritical fluids^45^ in the Chizhuangh eclogites (ca 30 km SSW of the sampled area) confirms the mobility of these fluids and their ability to transport elements out of source lithology.

### Implications for subduction geodynamics

**Figure 4:**
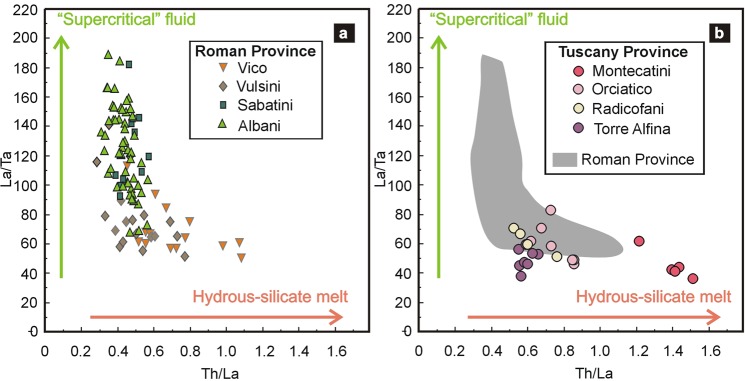
Th/La vs. La/Ta variations for ultrapotassic lavas. **(a)** Lavas from the Roman Province (Italy). **(b)** Lavas from the Tuscany Province. For comparison, lavas from Roman Province (Fig. 4a) are also reported. From the datasets^47^ (http://georoc.mpch-mainz.gwdg.de/georoc/), only analyses having MgO > 4 wt%, LOI < 4 wt%, and Na_2_O/K_2_O < 8 have been plotted in order to consider the most primitive rocks^46,47^.

Present work reports the first, natural evidence that UH*P* “supercritical” fluids released from subducting sediments along an intermediate geothermal gradient can be efficient metasomatic agents as HS melts. It also shows that, regardless of the source lithology, “supercritical” fluids have distinct fractionation patterns for selected elements. The raising question is if the orogenic/post-orogenic magmatism generated by processes involving metasomatic addition to the source mantle wedge by “supercritical” fluids or HS melts^1–7^ could preserve evidence of the nature of the agent, with consequences on the geothermal gradient of their source and, hence, on the geodynamic evolution of the area (e.g., subduction angle). We discuss, as an example, the Th/La vs. La/Ta fractionation in Plio-Quaternary potassic-ultrapotassic magmas from the Roman and Tuscany Provinces (Italy). The Roman magmas are interpreted as generated by partial melting of a carbonate-amphibole-phlogopite-bearing mantle metasomatized by HS melts or supercritical fluids released by carbonate-silicic sediments along a cold subduction gradient^46^. In Fig. 4a, the potassic-ultrapotassic magma compositions from Colli Albani, Sabatini, Vico, and Vulsini volcanic complexes^47^ are plotted in a Th/La vs. La/Ta diagram. Data for each volcanic complex are relatively gathered and distributed either along the fractionation trend defined by “supercritical” fluids (Colli Albani and Sabatini), or along that of HS melts (Vico and Vulsini). Such a distribution suggests that the metasomatic agent of the mantle source was a “supercritical” fluid, released at increasing temperature, that gradually changed its element fractionation to become indistinguishable from an HS melt. A similar evolution could fit with the progressive rise of isotherms at mantle depth by Adria roll-back^7^. Conversely, potassic-ultrapotassic magmas generated in a mantle source metasomatized by HS melts released along warmer subduction gradients would show constant low La/Ta and variable Th/La ratios. This is the case of the lamproitic magmas from the Tuscany Province (Italy; Fig. 4b) generated by melting of a phlogopite-bearing mantle at shallower depths^46^.

## Methods

### Raman micro-spectroscopy

Raman spectra and maps were acquired using the integrated micro/macro-Raman LABRAM HRVIS (Horiba Jobin Yvon Instruments) of the Interdepartmental Center “G. Scansetti” (Department of Earth Sciences, Universita di Torino, Italy), equipped with a computer-controlled, automated X–Y mapping stage. Excitation line at 532 nm (solid-state Nd laser and 80 mW of emission power) was used, with Edge filter and a grating of 600 grooves/mm. Calibration was performed using the 521 cm^−1^ Si band. Two maps of 11 μm × 14.5 μm, with steps of 1 μm and a laser spot of 1 μm, were acquired. The first, in the range 50–1750 cm^−1^, was acquired by one accumulation of 2 s for each step; the latter, in the range 2600–4300 cm^−1^, was acquired by one accumulation of 5 s for each step.

### Calculated major-element chemical composition

The major-element composition of the fluid was obtained by averaging the vol% of daughter minerals and liquid phase/empty spaces in 6 MSI’s, and considering their densities^34,49^ and compositions, following the method previously proposed for these rocks^35^. The associated errors in resulting fluid major-element composition (Supplementary table 1) were propagated by Monte-Carlo approach considering a 20%, 10% and 5% of errors in the estimation of the relative volume, absolute density, and chemical composition respectively, of single daughter phases within each MSI.

### LA-ICP-MS

*In situ* trace-element analysis of MSI’s and hosting kyanite were performed on doubly-polished 100-µm thick sections by using the LA-ICP-MS installed at the Department of Physics and Geology, Università di Perugia. The instrumentation consisted of a New Wave UP213 frequency-quintupled Nd:YAG laser ablation system coupled with a Thermo Fisher Scientific X series quadrupole-based ICP-MS. All LA-ICP-MS measurements were carried out using time-resolved analysis operating in a peak-jumping mode. Each analysis consisted of ~50 s of measuring the instrumental background – i.e. analysis of the carrier gas with no laser ablation – followed by ~60–100 s of data acquisition with the laser firing on the sample. The laser-beam diameter, the repetition rate and the laser energy density were fixed to 40 µm, 10 Hz and ~10 J/cm^2^ respectively. Helium was utilized as a carrier gas to enhance the transport efficiency of ablated aerosol^50^. The helium carrier exiting the ablation cell was mixed with argon make-up gas before entering the ICP torch to maintain a stable and optimum excitation condition. The LA-ICP-MS system was optimized for dry plasma conditions prior to each analytical session on a continuous linear ablation of NIST SRM612 glass standard by maximizing the signals for selected masses (La^+^ and Th^+^), minimizing oxide formation by reducing the ThO^+^/Th^+^ ratio below 0.5%, and maintaining the U^+^/Th^+^ ratio close to 1 (see also. Supplementary Fig. 8).

Two main LA-ICP-MS analytical protocols had been adopted here: (i) Ky-host mineral analysis on the sample surface, and (ii) MSI analysis in the depth of the sample.

For Ky-host mineral analysis (i), the time-resolved spectra were characterized by ~50 s of gas background followed by ~60 s signal related to the mineral phase. External calibration was performed using NIST SRM610 and SRM612 glasses standards in conjunction with internal standardization using ^29^Si, previously determined by WDS electron microprobe^51^. Data reduction was performed using the Glitter software^52^. The US Geological Survey (USGS) reference standard BCR2G (a fused glass of the Columbia River Basalt) was analyzed in each analytical run as quality control in order to assess the accuracy and the reproducibility of the analyses. Precision and accuracies are better than 10% for all the analyzed elements^53,54^. The analyses of quality controls utilized in the present study are reported in Supplementary table 2.

Unexposed MSI (10–35 µm in diameter) trace-element determinations (ii) was performed with a two-step procedure^55^. The first step consisted in the determination of the cumulative Ky-host plus MSI trace-element content. To do this, the gas background is acquired for ~50 s (segment A in Supplementary Fig. 8), then the laser is turned on. At the beginning of the ablation, the mass spectrum was only characterized by the signals related to the Ky-host (segment B in Supplementary Fig. 8). When the laser beam reached the depth of MS-inclusion, a signal characterized by the contribution of both the Ky-host and the MS-inclusion was acquired (segment C in Supplementary Fig. 8). At the end of the MSI, ablation signals returned to the Ky-host values (segment D in Supplementary Fig. 8). Raw signals of the segment C (Supplementary Fig. 8) were reduced to concentration values (*C*_*i*_^*MSI*+*Ky host*^) using ^29^Si as internal standard^51^. The concentration of ^29^Si was calculated utilizing the following relation:

1 $${C}_{Si{O}_{2}}^{MSI+Kyhost}={C}_{Si{O}_{2}}^{MSI}x+{C}_{Si{O}_{2}}^{Kyhost}(1-x)$$

Where ${C}_{Si{O}_{2}}^{MSI}$ and ${C}_{Si{O}_{2}}^{Kyhost}$ are the SiO_2_ concentration of the MSI’s reported in Table 1, and the kyanite SiO_2_ concentration analyzed by WDS, respectively. Finally, the relative mass fraction *x* of the MSI (*m*^*MSI*^) over the total ablated mass (*m*^*MSI*+*Ky host*^) was estimated as follows^55^

2 $$x=\frac{{m}^{MSI}}{{m}^{MSI+Kyhost}}=\frac{{V}^{MSI}\cdot {\rho }^{MSI}}{{V}^{MSI}\cdot {\rho }^{MSI}+{V}^{Kyhost}\cdot {\rho }^{Kyhost}}$$

where *V* and *ρ* correspond to volume and density values, respectively^55^.

In order to estimate *V*^*MSI*^, each MSI, having a negative crystal shape, was approximated as a parallelepiped where the x-, y- and z-axes are quantified by using an optical microscope. The volume of the ablated material during the acquisition of the segment C (Supplementary Fig. 8) was approximated as a cylinder having the diameter of the laser beam and a depth equal of the z-axis of the MS-inclusion. Trace-element composition of MSI were then estimated (step two) by correcting the obtained trace-element values for the contribution of the Ky-host mineral^55^. It simply consists in assuming the Ky-host plus MSI trace-element contents as the result of a mixing between the Ky-host and the MSI. The equation of mixing is then utilized in order to obtain the trace-element contents (*C*_*i*_^*MSI*^) of the MSI^55^

3 $${{C}_{i}}^{MSI}={{C}_{i}}^{Kyhost}-\frac{({C}_{i}^{Kyhost}-{C}_{i}^{MSI+Kyhost})}{x}$$

In the above equation *C*_*i*_^*Ky host*^ is the trace-element content of Ky-host, *C*_*i*_^*MSI*+*Ky host*^ is the concentration of the mixed analysis and *x* is the mass ratio defined in the Eq. (2). Results are reported in Supplementary table 2. Finally, the error propagation has been modelled using a Monte-Carlo approach considering the uncertainties reported in Supplementary table 1 and 2 for *C*_*i*_^*Ky host*^, *C*_*i*_^*MSI*+*Ky host*^, and *x* respectively. The resulting uncertainties are also reported in Supplementary Table 2 and plotted in Supplementary Fig. 9.

## Supplementary information

Supplementary information
