# Supplementary material for: Gradual and selective trace-element enrichment in slab-released fluids at sub-arc depths
Source: Sci Rep. 2019 Nov 8;9:16393. doi: 10.1038/s41598-019-52755-9 (PMC6841933; doi:10.1038/s41598-019-52755-9)
Supplement: Supplementary file 1 — Supplementary information [file 41598_2019_52755_MOESM1_ESM.doc]

**Gradual and selective trace-element enrichment in slab-released fluids at sub-arc depths**

Simona Ferrando^1*^, Maurizio Petrelli^2^, Maria Luce. Frezzotti ^3*^

^1^Department of Earth Sciences, Università di Torino, Via Valperga Caluso 35, 10125 Torino, Italy

^2^Department of Physics and Geology, Università di Perugia, Perugia, Piazza Università 1, 06100 Perugia, Italy

^3^Department of Earth and Environmental Sciences, Università di Milano-Bicocca, Piazza della Scienza 4, 20126 Milano, Italy.

e-mails: simona.ferrando@unito.it; maria.frezzotti@unimib.it

**SUPPLEMENTARY RESULTS**

**Geology of Sulu terrane and sample description.** The Qinling-Dabie-Sulu orogen (China, Supplementary Fig. 1a) was formed by Triassic subduction and collision of Yangtze craton beneath Sino-Korean craton. The Sulu terrane consists of ultrahigh-pressure (UH*P)*, high pressure (H*P)* metamorphic, and migmatitic basements^1^, Cretaceous granitic plutons, and Mesozoic and more recent sediments^2^. In southern Sulu terrane (Supplementary Fig. 1b), the UH*P* Unit consists of orthogneiss including layers and/or boudins of paragneiss, eclogite variably retrogressed to amphibolite, ultramafic rocks, quartzite and marble^1,3^. Peak metamorphic conditions, dated at 235-225 Ma^4^, are primarily estimated at *T*=730-890°C and *P*= 3.5-4.5 GPa^1,5-8^ (Supplementary Fig. 2a). Recently, pseudosection modelling seems to suggest lower *P-T* conditions (660-690°C and 3.1-3.3 GPa)^9^. Extremely low δ^18^O and δD values preserved in the Donghai lithologies suggest that their protolith interacted with fluids in a hydrothermal meteoric water system during a period of cold climate, and that the subsequent evolution occurred in a relatively closed system^10,11^.

Two types of kyanite-quartzite were identified in the Donghai area^12^. “Type 1” kyanite-quartzite constitutes coarse-grained UH*P* veins crosscutting the main foliation of the eclogites. It mainly consists of quartz ± omphacite (or jadeite) ± kyanite ± allanite ± zoisite ± rutile ± garnet^13^. “Type 2” kyanite-quartzite, to which belongs the studied sample, occurs within gneiss and it is weakly foliated along the main foliation (S_p_; Supplementary Fig. 2b). It shows an UH*P* (2.9-3.9 GPa, 700-830 °C; 235-225 Ma^4,5,7^) mineral assemblage consisting of coesite (now polycrystalline quartz), porphyroblastic and neoblastic kyanite, and minor relict phengite (stages A_2_ and B_1_ in Supplementary Fig. 2). Pre-S_p_ dimensionally-oriented porphyroblastic kyanite grows at the expense of phengite (Supplementary Fig. 2c) at UH*P* prograde-to-peak conditions (stages A_1_ and A_2_ in Supplementary Fig. 2b). The core (Ky-Ia) locally shows rare relics of a folded S_p-1_ (Fig. 2c^7^) and, such as the rim (Ky-Ib), includes relict phengite and (former) coesite. Kyanite porphyroblasts are locally crowded by fluid inclusions (Supplementary Fig. 2e). Syn-S_p_ crystallographically oriented neoblastic kyanite (Ky-II) grows during early decompression^7^ (Supplementary Fig. 2b). It never includes phengite relics, (former) coesite and fluid inclusions (Fig. 2^7^) and represents the last generation of kyanite. “Type 2” kyanite-quartzite contains scarce retrograde minerals (OH-rich topaz, muscovite, paragonite, pyrophyllite^7,14,15^; Supplementary Fig. 2b) and lacks evidence for the H*P* partial melting locally observed in the Dabie-Sulu terrane^16^. Accessory minerals are rutile, zircon, pyrite, barite, monazite (Supplementary Fig. 2f) and apatite. Both petrographic and isotopic data point to a sedimentary protolith, possibly a clay-rich quartz-arenite^3,4,7^.

The studied sample (RPC547) was collected at Hushan (35.29139N; 118.531206E), S of Qinglongshan (Donghai area; Supplementary Fig. 1b), from a trench 3m deep to the SW of a ridge consisting of gneiss. With other samples, it was selected to constrain metamorphic and fluid evolution of this lithology^7,17^ Supplementary Fig. 2b). It has been selected for present study because: i) it lacks relict phengite in rock matrix, suggesting complete phengite destabilization ; ii) it contains a lower amount of MSI’s with respect to the other samples (Supplementary Fig. 2e) which makes easier to select isolated inclusions for LA-ICP-MS analysis (see below); iii) the very limited chemical variations (e.g. an increase in Pb and La) measured from core (Ky-Ia) to rim (Ky-Ib) of porphyroblastic kyanite hosting MSI’s help to locate them from a microstructural point of view (Supplementary Fig. 2d; Supplementary Table 2).

**Multiphase-solid inclusions: petrography and major-element composition.** Studied fluid inclusions occur as surprisingly abundant primary multiphase-solid inclusions (MSI’s) in UH*P* core and rim of porphyroblastic kyanite (Ky-Ia and Ky-Ib in Supplementary Fig. 3a). They are evenly distributed and have constant dimensions ranging from 5 to 30 μm in length (Supplementary Fig. 3a-b). MSI’s apparently preserved from relevant post-trapping modifications have negative-crystal shape and are filled by an aggregate of muscovite, paragonite, K-Na-hydrous sulfate, anhydrite, carbonates, minor pyrite, barite and corundum, and by an aqueous fluid (Supplementary Fig. 3d-k). Solid and fluid phases show relatively constant proportions and represent, respectively, the daughter minerals precipitated from the trapped liquid and the residual fluid^18^. More rarely, MSI’s show strong evidence for post-trapping modifications, such as irregular contours and/or offshoots departing from the inclusion corners (Supplementary Fig. 3c). In these MSI’s, the volume of the aqueous fluid is lower than that in the most preserved ones, and some daughter minerals show evidence for retrograde hydration reactions (e.g., diaspore around relict corundum; Fig. 2c^17^). Although water diffusion in the host mineral^18^ cannot be excluded during early UH*P*-H*P* decompression, selected MSI’s do not show evidence for change in fluid chemistry by interaction with host kyanite, neither kyanite shows evidence of incipient hydration and reaction. The MSI’s showing star-shaped contours (Supplementary Fig. 3b) and retrograde hydration of the host mineral (e.g., topaz at the contact between host kyanite and MSI; Fig. 3b^7^) have been carefully discarded. More rarely, both preserved an modified MSI’s contains incidentally-trapped minerals (zircon, rutile) that are not precipitated from the trapped fluid, but belong to the rock-mineral assemblage in equilibrium with the fluid.

Multiphase-solid inclusions represent aqueous fluids containing Al, Si, S, Ca, K, Na and minor dissolved CO_2_. The calculated average fluid composition for studied sample RPC547 has been obtained from the composition of 6 MSI’s, not analyzed previuosly^7,17^, reported in Supplementary Table 1. With respect to composition calculated averaging data from three kyanite quartzite samples^17^, that obtained in the present study has lower SiO_2_ and Al_2_O_3_ [with similar SiO_2_/(SiO_2_+Al_2_O_3_) ratio], lower alkalis [but a similar K_2_O/(Na_2_O+K_2_O) ratio], higher CaO, FeO, MgO, CO_2_, SO_3_, and lower H_2_O. The original water content should have been considerably higher, in the order of 40–60 wt%, but was partly lost by passive H_2_O diffusion from inclusions, even from the most preserved ones, during retrogression^18,19^. Previous works^7,17^ demonstrate that MSI’s from Sulu kyanite quartzite are remnants of an alkali-alumino-silicate aqueous solution, with composition intermediate between an aqueous fluid and a hydrous-silicate melt, generated by dehydration reactions involving phengite near the UH*P* metamorphic peak (Supplementary Fig. 2a-b), i.e. near or above the second critical end-point of the system^20^.

**Multiphase-solid inclusions selected for LA-ICP-MS analyses.** Multiphase-solid inclusions selected for LA-ICP-MS analyses (Supplementary Table 2; Figs. 1-3; Supplementary Figs. 4, 6-9) show the following properties (Figs. 1a-c): a) they are located near the sample surface (ca < 10 µm) in order to have the best analytical signal; b) they are isolated to exclude a mixed contribution from more than one inclusion during the analysis; c) they have sizes similar, but not higher, than the laser-beam diameter (i.e., ca 30-40 µm); d) they lack evidence of post-trapping chemical modifications; e) they do not contain incidentally-trapped minerals based on optical observations.

In the studied sample, only six MSI’s comply these pre-requisites, as commonly occurs in trace-element studies on natural UH*P* fluid inclusions^21-25^. The microstructural position of the analyzed MSI’s with respect to the stages of growth of porphyroblastic kyanite has been constrained by microscopic observations and by trace-element variations in kyanite (Supplementary Fig. 2d). MSI2 and3 (Fig. 1a) are located at the same focus in prograde Ky-Ia inner core. MSI8, 22, 23 and 7 (Figs. 1b-c) are located at different depths of another kyanite porphyroblast full of inclusions (Supplementary Fig. 3a). MSI 7 is the most external (i.e. trapped in peak Ky-Ib rim), whereas MSI8, 22 and 23 are slightly deeper and more internal (i.e. trapped in prograde Ky-Ia outer core). This implies that the collected data are indicative for a fluid trapped during UHP prograde-to-peak increase in temperature and, possibly, pressure (stages A1-A2 in Supplementary Fig. 2b).

Trace-element compositions of MSI’s reported in Supplementary Table 2 are recalculated by the mixed (MSI + host kyanite) data obtained by in situ LA-ICP-MS analyses (see the section Methods). Because trace-elements are usually highly incompatible in kyanite (Supplementary Fig. 5), their estimate in MSI’s can be considered reliable.

SUPPLEMENTARY REFERENCES

1. Zhang, R.Y. *et al.* Petrology of ultrahigh-pressure rocks from the southern Su-Lu region, eastern China. *Journal of Metamorphic Geology* **13**, 659-675 (1995).
2. Wallis, S., Enami, M. & Banno, S. The Sulu UHP Terrane - a review of the petrology and structural geology. *International Geology Review* **41**, 906-920 (1999).
3. Zhang, Z., Xu, Z. & Xu, H. Petrology of ultrahigh-pressure eclogites from the ZK703 drillhole in the Donghai, eastern China. *Lithos* **52,** 35-50 (2000)
4. Liu, F.L. & Liou, J.G. Zircon as the best mineral for P–T-time history of UHP metamorphism: a review on mineral inclusions and U\Pb SHRIMP ages of zircons from the Dabie–Sulu UHP rocks. *Journal of Asian Earth Sciences* **40**, 1–39 (2011).
5. Zhang, Z., Xiao, Y., Liu, F., Liou, J.G. & Hoefs, J. Petrogenesis of UHP metamorphic rocks from Qinglongshan, southern Sulu, east-central China. *Lithos* **81**, 189–207 (2005)
6. Ferrando, S., Frezzotti, M. L., Dallai, L. & Compagnoni, R. Fluid-rock interaction in UHP phengite-kyanite-epidote eclogite from the Sulu orogen, Eastern China. *International Geology Review* **47**, 750-774 (2005).
7. Frezzotti, M. L., Ferrando, S., Dallai, L. & Compagnoni, R. Intermediate alkali-alumino-silicate aqueous solutions released by deeply subducted continental crust: fluid evolution in UHP OH-rich topaz-kyanite quartzites from Donghai (Sulu, China). *Journal of Petrology* **48**, 1219-1241 (2007).
8. Wang, L. *et al.* Partial melting of deeply subducted eclogite from the Sulu orogen in China. *Nature Communications* **5**, 5604, doi:10.1038/ncomms6604 (2014).
9. Li, Z. *et al.* Metamorphic P-T path differences between the two UHP terranes of Sulu orogen, Eastern China: petrologic comparison between eclogites from Donghai and Rongcheng. *Journal of Earth Science* **29**, 1151-1166 (2018).
10. Rumble, D. III & Yui, T.-F. The Qinglongshan oxygen and hydrogen isotope anomaly near Donghai in Jiangsu Province, China. *Geochimica et Cosmochimica Acta* **62**, 3307-3321 (1998).
11. Zheng, Y.-F., Fu, B., Gong, B. & Li, L. Stable isotope geochemistry of ultrahigh pressure metamorphic rocks from the Dabie-Sulu orogen in China: implications for geodynamics and fluid regime. *Earth-Science Reviews* **62**, 105–161 (2003)
12. Zhang, R.Y., Liou, J.G. & Ye, K. in *Ultrahigh-pressure Metamorphic Rocks in the Dabieshan-Sulu Region, China* (ed. B. Cong) 49-68 (Beijing, Science Press).
13. Zhang, Z. M. *et al.* Fluid in deeply subducted continental crust: petrology, mineral chemistry and fluid inclusion of UHP metamorphic veins from the Sulu orogen, eastern China. *Geochimica et Cosmochimica Acta* **72**, 3200-3228 (2008).
14. Zhang, R. Y., Liou, J. G. & Shu, J. F. Hydroxyl-rich topaz in high-pressure and ultrahigh-pressure kyanite quartzites, with retrograde woodhouseite, from the Sulu terrane, eastern China. . *American Mineralogist* **87**, 445-453 (2002).
15. Alberico, A., Ferrando, S., Ivaldi, G. & Ferraris, G. X-ray single-crystal structure refinement of an OH-rich topaz from Sulu UHP terrane (Eastern China). Structural foundation of the correlation between cell parameters and fluorine content. *European Journal of Mineralogy* **15**, 875-881 (2003).
16. Zheng, Y. F., Xia, Q. X., Chen, R. X. & Gao, X. Y. Partial melting, fluid supercriticality and element mobility in ultrahigh-pressure metamorphic rocks during continental collision. *Earth-Science Reviews* **107**, 342-374 (2011).
17. Ferrando, S., Frezzotti, M. L., Dallai, L. & Compagnoni, R. Multiphase solid inclusions in UHP rocks (Su-Lu, China): remnants of supercritical silicate-rich aqueous fluids released during continental subduction. *Chemical Geology* **223**, 68-81 (2005).
18. Frezzotti, M. L. & Ferrando, S. The chemical behavior of fluids released during deep subduction based on fluid inclusions. *American Mineralogist* **100**, 352-377 (2015).
19. Frezzotti, M. L., Ferrando, S., Tecce, F. & Castelli, D. Water content and nature of solutes in shallow-mantle fluids from fluid inclusions. *Earth and Planetary Science Letters*, **351-352**, 70-83 (2012).
20. Hermann, J., Zheng, Y. F. & Rubatto, D. Deep fluids in subducted continental crust. *Elements* **9**, 281-287 (2013).
21. Scambelluri, M., Bottazzi, P., Trommsdorff, V., Vannucci, R., Hermann, J., Gomez-Pugnaire, M. T. & Lòpez-Sànchez Vizcaìno, V. Incompatible element-rich fluids released by antigorite breakdown in deeply subducted mantle. *Earth and Planetary Science Letters*, **192**, 457-470 (2001).
22. Malaspina, N., Hermann, J., Scambelluri, M. & Compagnoni, R. Polyphase inclusions in garnet-orthopyroxenite (Dabie Shan, China) as monitors for metasomatism and fluid-related trace element transfer in subduction zone peridotite. *Earth and Planetary Science Letters*, **249**, 173-187 (2006).
23. Malaspina, N., Hermann, J. & Scambelluri, M. Fluid/mineral interaction in UHP garnet peridotite. *Lithos*, **107**, 38-52 (2009).
24. Ferrando, S., Frezzotti, M. L., Petrelli, M. & Compagnoni, R. Metasomatism of continental crust during subduction: the UHP whiteschists from the Southern Dora-Maira Massif (Italian Western Alps). *Journal of Metamorphic Geology* **27**, 739-756 (2009).
25. Gao, X. Y., Zheng, Y. F., Chen, Y. X. & Hu, Z. Trace element composition of continentally subducted slab-derived melt: insight from multiphase solid inclusions in ultrahigh-pressure eclogite in the Dabie orogen. *Journal of Metamorphic Geology*, **31**, 453-468 (2013).
26. Zeng, L. S., Liang, F. H., Asimov, P. D., Chen, F. Y. & Chen, J. Partial melting of deeply subducted continental crust and the formation of quartzofeldspathic polyphase inclusions in the Sulu UHP eclogites. *Chinese Science Bulletin* **54**, 2580-2594, (2009).
27. Wang, W. *et al.* Petrological and geochronological constraints on the origin of HP and UHP kyanite-quartzites from the Sulu orogen, Eastern China. *Journal of Asian Earth Sciences* **2011**, 618-632 (2011).
28. Whitney, D. L. & Evans, B. W. Abbreviations for names of rock-forming minerals. *American Mineralogist* **95**, 185-187, (2010).
29. Hack, A. C. & Thompson, A. B. Density and viscosity of hydrous magmas and related fluids and their role in subduction zone processes. *Journal of Petrology* **52**, 1333-1362 (2011).
30. Spandler, C., Mavrogenes, J. A. & Hermann, J. Experimental constraints on element mobility from subducted sediments using high-P synthetic fluid/melt inclusions. *Chemical Geology* **239**, 228-249 (2007)
31. Kessel, R., Schmidt, M. W., Ulmer, P. & Pettke, T. Trace element signature of subduction-zone fluids, melts and supercritical liquids at 120-180 km depth. *Nature* **437**, 724-727 (2005).
32. Hermann, J. & Rubatto, D. Accessory phase control on the trace element signature of sediment melts in subduction zones. *Chemical Geology* **265**, 512-526 (2009).
33. Skora, S. & Blundy, J. D. High-pressure hydrous phase relations of Radiolarian clay and implications for the involvement of subducted sediment in arc magmatism. *Journal of Petrology* **51**, 2211-2243 (2010).
34. Carter, L. B., Skora, S., Blundy, J. D., De Hoog, J. C. M. & Elliott, T. An experimental study of trace element fluxes from subducted oceanic crust. *Journal of Petrology* **56**, 1585-1606 (2015).
35. Skora, S. *et al.* Hydrous phase relations and trace rlement partitioning behaviour in calcareous sediments at subduction-zone conditions. *Journal of Petrology* **56**, 953-980 (2015).
36. Tsay, A., Zajacz, Z., Ulmer, P. & Sanchez-Valle, C. Mobility of major and trace elements in the eclogite-fluid system and element fluxes upon slab dehydration. *Geochimica et Cosmochimica Acta* **198**, 70-91 (2017).


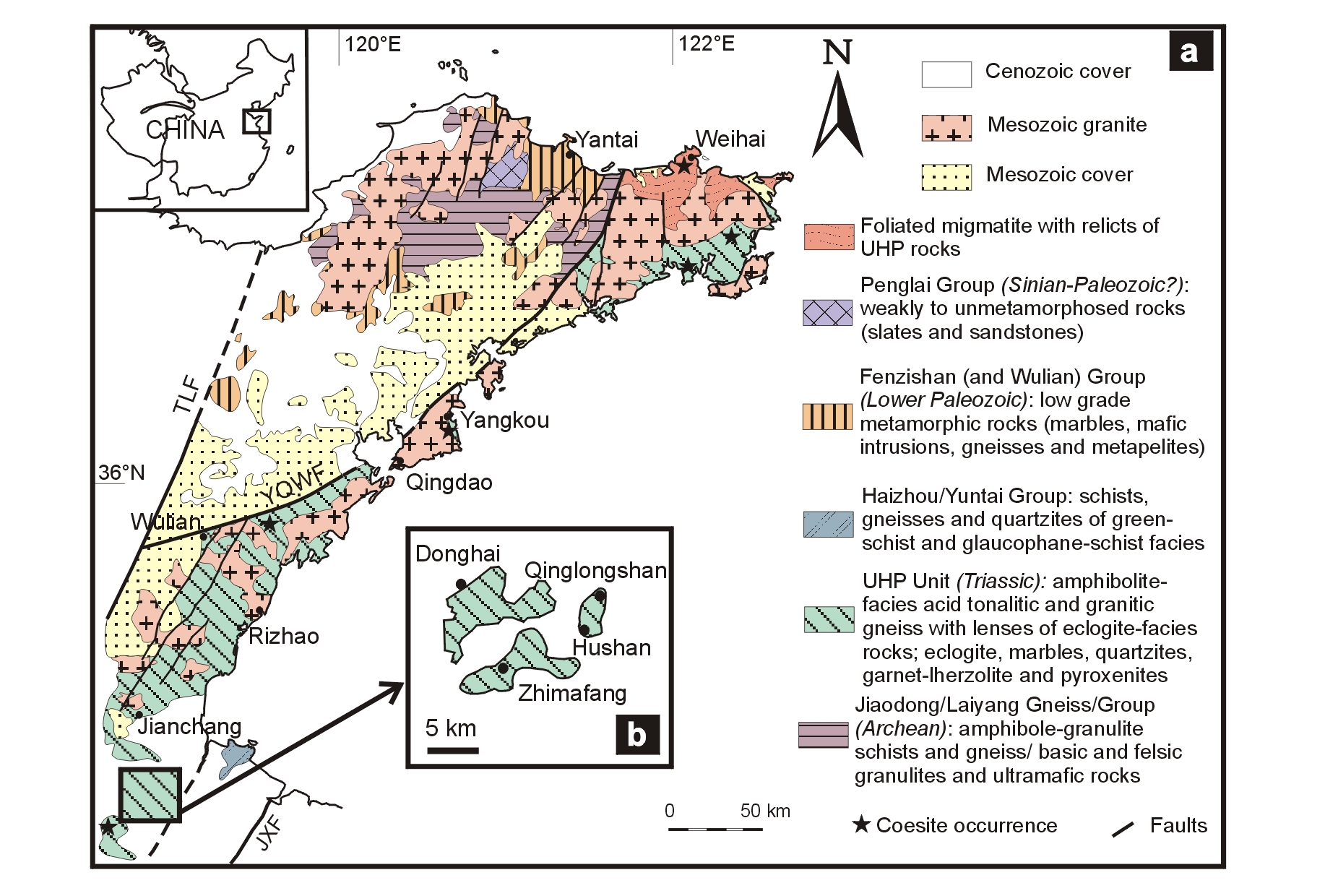


**Supplementary Figure 1. Tectono-metamorphic map of Sulu and sample location. (a)** Major tectonic Units and coesite occurrences^7^. Faults: TLF=Tan-Lu; JXF=Jianshan-Xiangshui; YQWF=Yantai-Qingdao-Wulian. **(b)** Enlargement of Donhai area and sample location^26-27^.


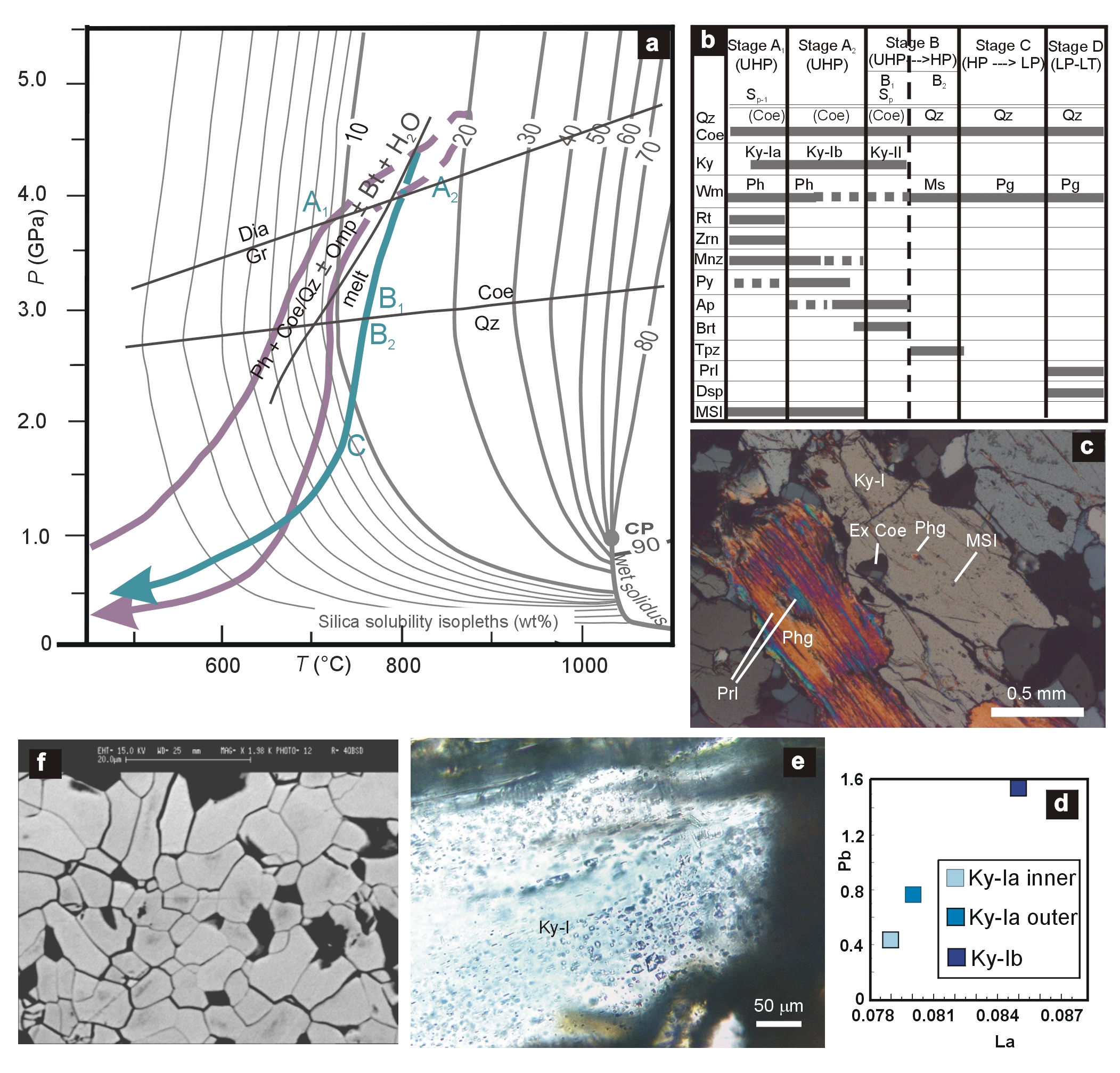


**Supplementary Figure 2. Petrography and metamorphic evolution of kyanite-quartzite.** **(a)** *P-T* path with stages A_2_-C (blue^7^), *P-T* path of Sulu UHP belt^8^, Ph^28^ break-down curve^16^, and SiO_2_ solubility isopleths and second critical end-point (CP) in the H_2_O-SiO_2_ system^29^. **(b)** Metamorphic evolution modified from^7^ (Wm=white mica). **(c)** Photomicrograph of relict Ph partly overgrown by porphyroblastic Ky-I (RPC545; crossed polarized light: XPL). **(d)** Trace-element chemical zoning of porphyroblastic kyanite (RPC547). **(e)** Photomicrograph showing the MSI distribution in porphyroblastic kyanite (RPC542; plane polarized light: PPL). **(f)** Back-scattered electron (BSE) image of an aggregate of monazite (RPC542).


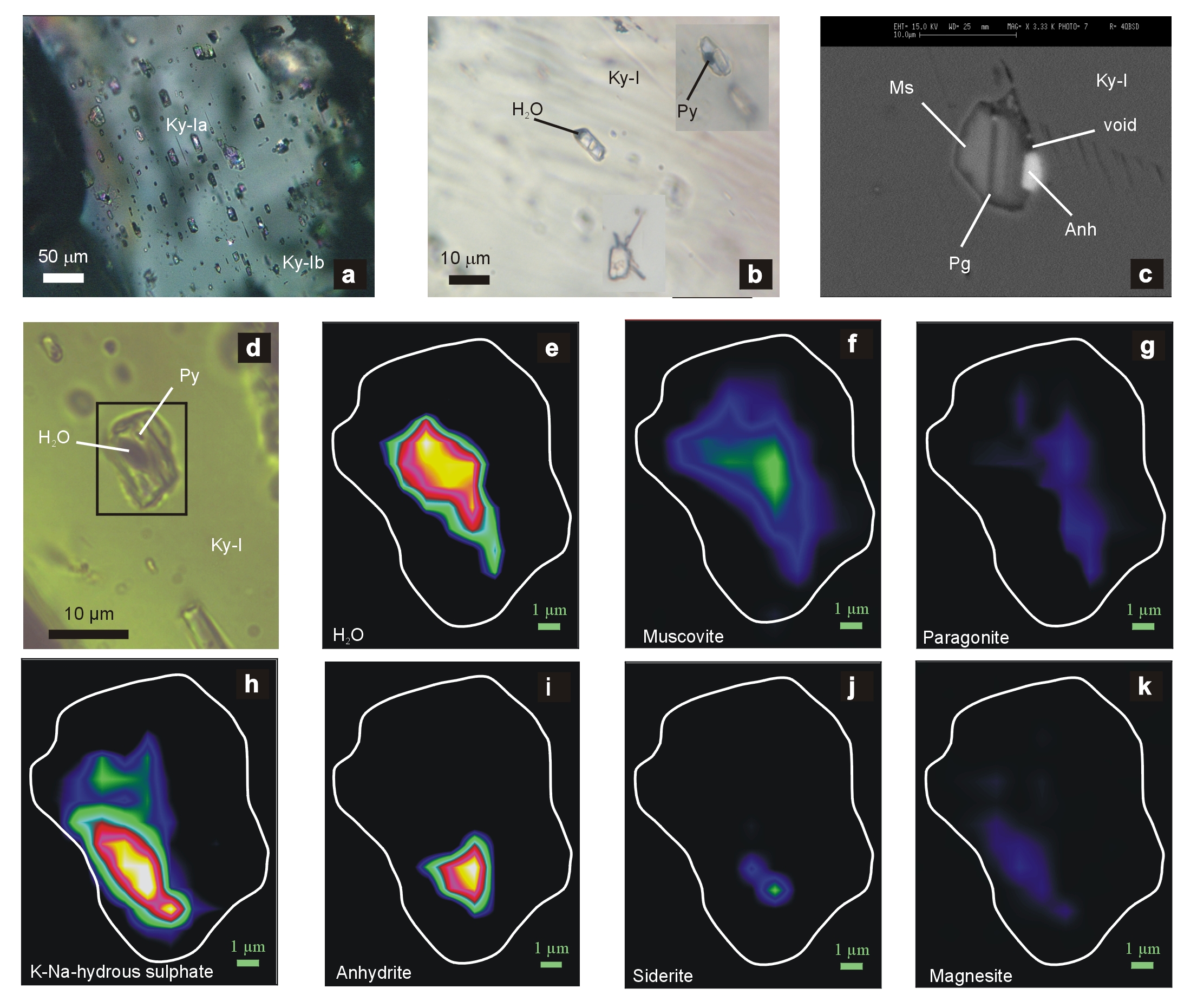


**Supplementary Figure 3. Multiphase-solid inclusions in porphyroblastic Ky-I.** **(a-b)**: Photomicrograph of **(a)** primary MSI’s in kyanite, and **(b)** preserved and decrepitated MSI’s (plane polarized light: PPL). **(c)** Back-scattered electron image of a MSI showing the typical oriented association of daughter-minerals and a cavity. **(d)** Photomicrograph of the mapped MSI (PPL). **(e-k)** Raman spectral images showing distribution of water (3500 cm^-1^), muscovite (268 cm^-1^), paragonite (218 cm^-1^), K-Na-hydrous sulfate (3487 cm^-1^), anhydrite (1018 cm^-1^), siderite (1095 cm^-1^), and magnesite (1093 cm^-1^), respectively. The color intensity (from black to white) reflects the relative increase in the intensity of the Raman band.


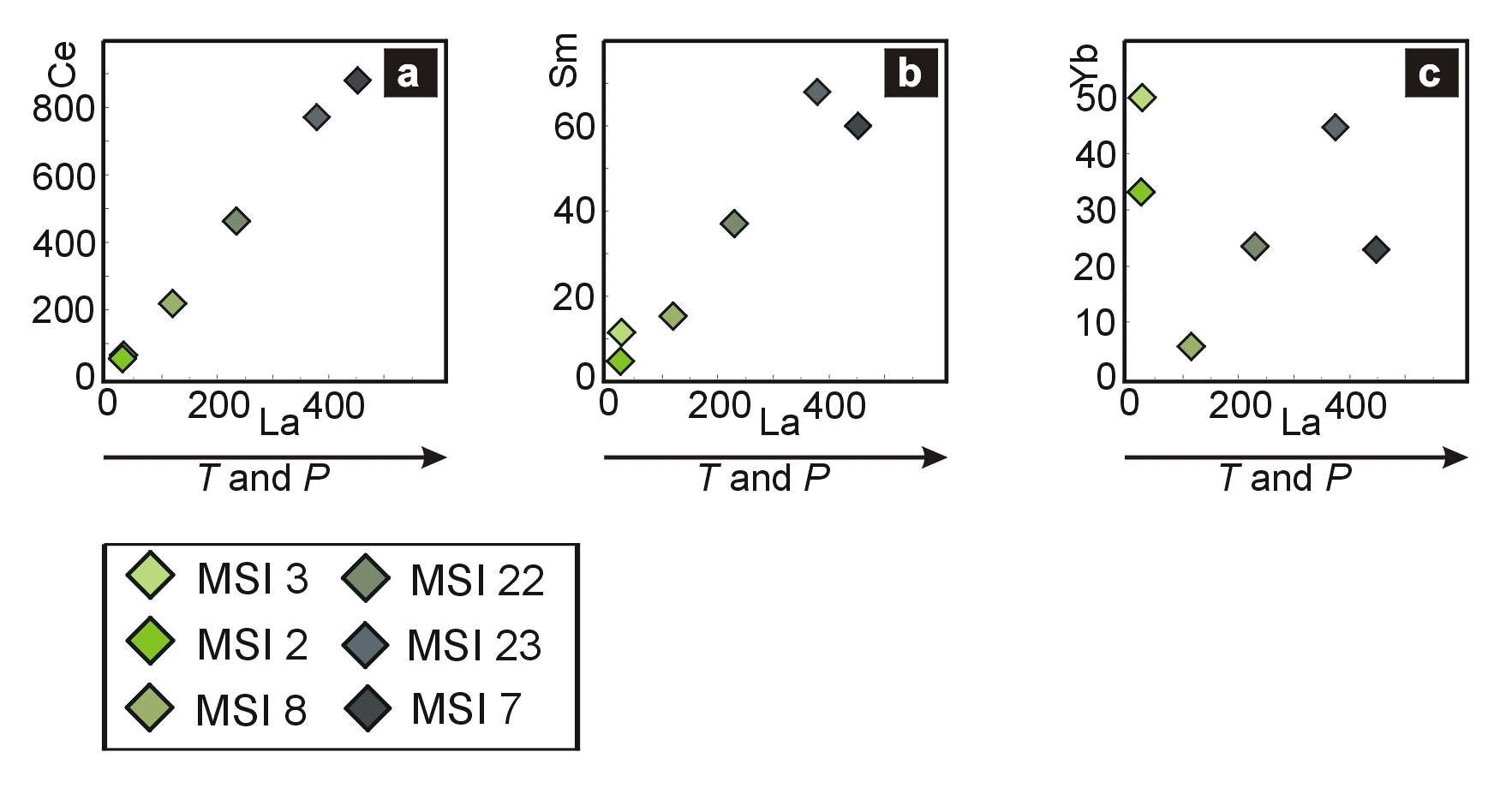


**Supplementary Figure 4. REE concentrations (in ppm) with respect to La.** The content in LREE and MREE, in contrast with that in HREE, progressively increases from MSI inclusions located in prograde Ky-Ia inner core (MSI2 and 3) to that located in peak Ky-Ib rim (MSI7).


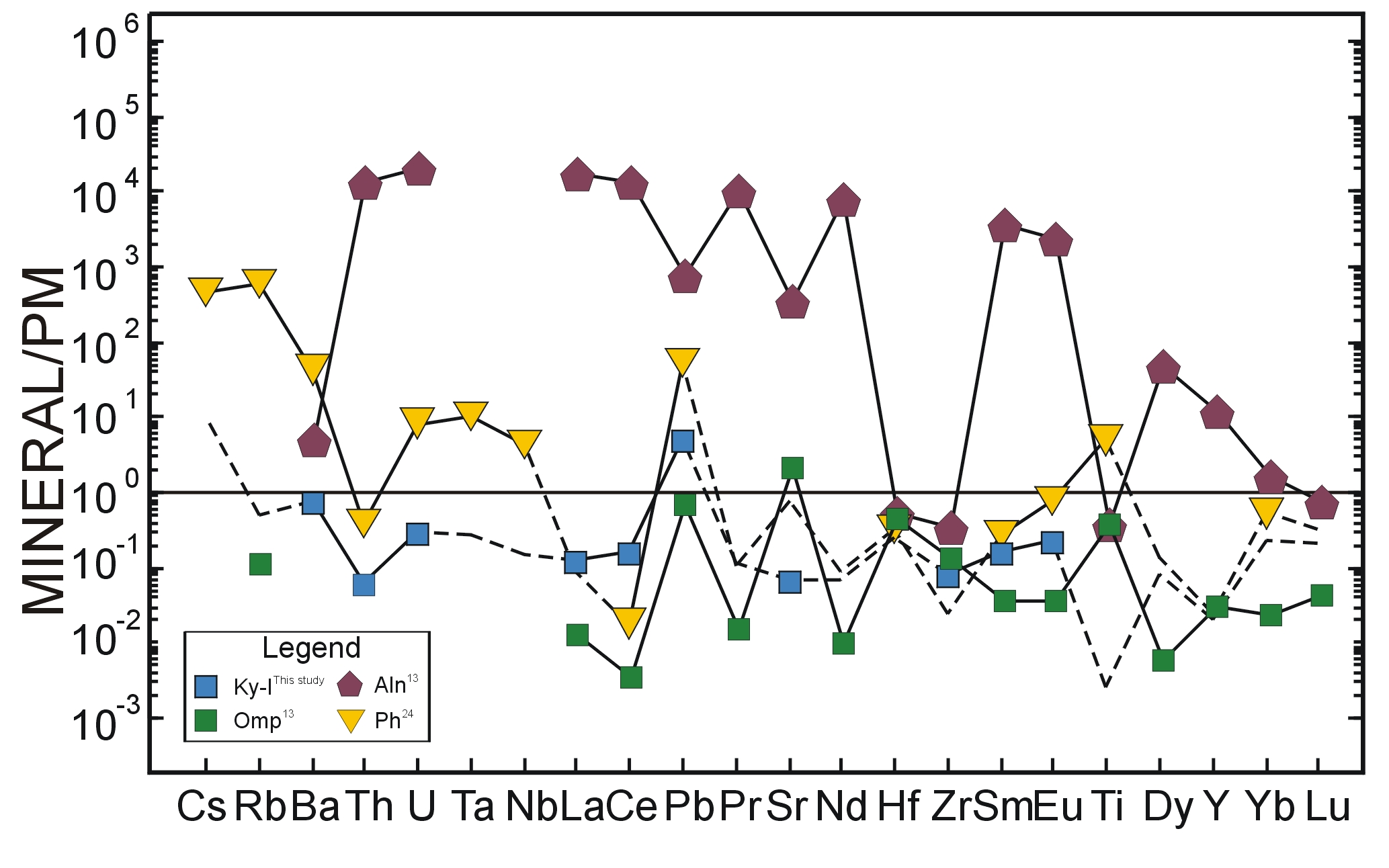


**Supplementary Figure 5. Trace-element patterns of minerals relevant for the present study.** Data from present study and literature^13,24^.


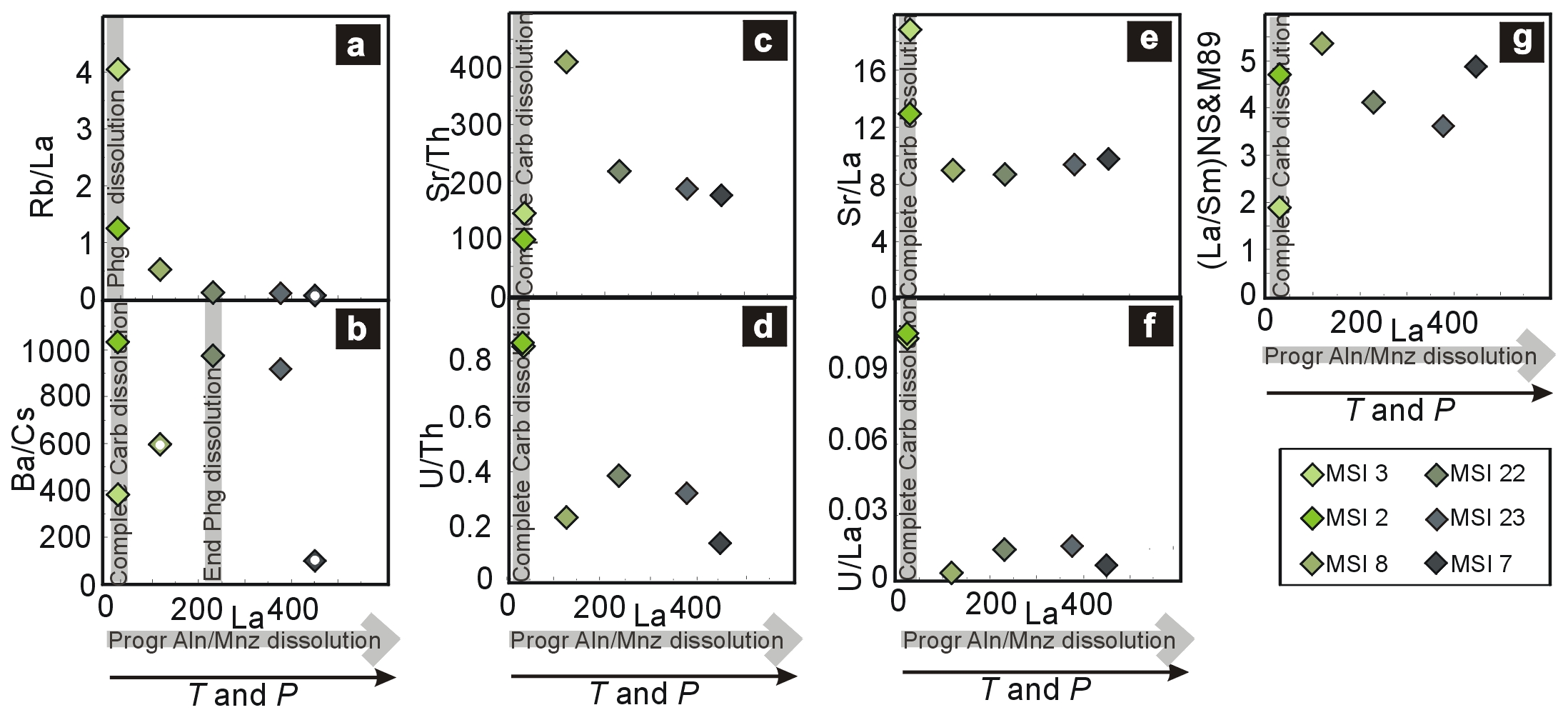


**Supplementary Figure 6. Trace-element ratios in MSI’s with respect to La (in ppm).** **(a-g)** Diagrams showing the variation in trace-element ratios produced by progressive/complete dissolution of carbonate and phengite during UH*P* prograde-to-peak evolution. The white dot refers to ratios in which element detection limit has been used as maxim element content.


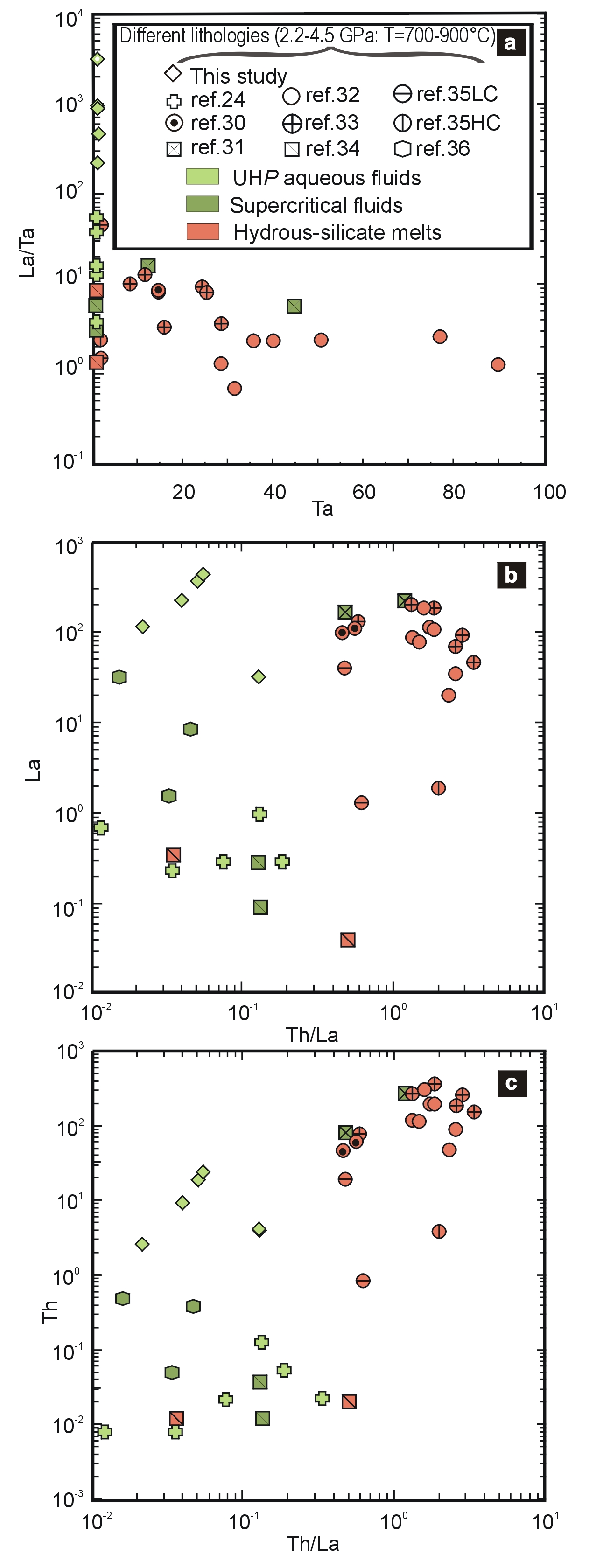


**Supplementary Figure 7. Comparison among trace-element ratios from natural aqueous fluids and from experimental supercritical fluids and hydrous-silicate melts released by different lithologies at high *P-T* conditions (2.2-4.5 GPa; 700-900°C).** **(a-c)** Data from present study and the literature^24,30-36^. The white dot refers to ratios in which Ta detection limit has been used as maxim Ta content. MSI2 was not plotted in Fig. 7a because its Ta content is disguised by the presence of a mineral not precipitated from the fluid (see text and Fig. 1d).

**Supplementary Figure 8. Illustrative example of MSI transient data acquisition and reduction.** The figure reports the intensities (in count per second, cps) of selected analyses vs. time (in seconds, s) acquired by LA-ICP-MS. Also, it reports how the different signal segments were selected: A) gas background, B) Ky-host before the MSI, C) mixed signal of Ky-host + MSI, D) Ky-host after the MSI

**Supplementary Figure 9. Trace-element patterns with errors.** Trace-element pattern of each analyzed MSI (see also Fig. 1d) with error-bars.
